# Ultra-processed foods – a scoping review for Nordic Nutrition Recommendations 2023

**DOI:** 10.29219/fnr.v68.10616

**Published:** 2024-04-24

**Authors:** Filippa Juul, Elling Bere

**Affiliations:** 1 School of Global Public Health, New York University, New York, NY, USA; 2 Department of Sports Science and Physical Education, University of Agder, Kristiansand, Norway; 3 Center for Epidemiological Studies in Health and Nutrition, School of Public Health, University of São Paulo, São Paulo, Brazil

**Keywords:** ultra-processed foods, NOVA, processing, nutrition recommendations

## Abstract

Ultra-processed foods (UPFs) are increasingly consumed worldwide and have been linked to several chronic diseases. This paper aims to describe the totality of the available evidence regarding UPFs in relation to health-related outcomes as a basis for setting food-based dietary guidelines for the Nordic Nutrition Recommendations 2023. Systematic literature searches were conducted to identify systematic reviews, meta-analyses, randomized controlled trials (RCTs), and prospective cohort studies examining the association between UPF intake and non-communicable diseases or mortality. A total of 12 systematic reviews (including five meta-analyses) and 44 original research studies (43 prospective cohort studies and one RCT) were included. All original research studies were deemed to be of good methodological quality. The current evidence supports that greater consumption of UPFs is associated with weight gain and increased risk of obesity, cardiovascular disease, type 2 diabetes, and all-cause mortality. The available literature also supports an association between UPFs and hypertension, cancer, and depression; however, the limited number of studies and subjects investigated preclude strong conclusions. Due to the highly diverse nature of UPFs, additional studies are warranted, with special emphasis on disentangling mediating mechanisms, whether nutritional or non-nutrient based. Nevertheless, the available evidence regarding UPFs in relation to weight gain, CVD, type 2 diabetes, and all-cause mortality is considered strong enough to support dietary recommendations to limit their consumption.

## Popular scientific summary

- Ultra processed foods (UPF) are defined within the NOVA framework as formulations of ingredients, mostly of exclusive industrial use, that result from a series of industrial processes
- Diets high in UPFs are consistently associated with weight gain, cardiovascular disease, type 2 diabetes, and all-cause mortality in high quality prospective cohort studies
- Experimental evidence supports that diets based on UPF lead to excessive energy intakes and weight gain in the short term
- Limiting the intake of UPFs may reduce the risk of premature mortality, obesity, cardiovascular disease and type 2 diabetes

Nutrition research, dietary guidelines, and national and international policies have historically focused on nutrients rather than foods and how these are processed (1). Food-based dietary guidelines (FBDG) entered the scene gradually and is today implemented in at least 96 countries worldwide (2). Recently, however, there is a rapidly growing scientific interest in highly processed or so-called ultra-processed foods (UPFs), as accumulating evidence links their consumption to poor diet quality and chronic disease outcomes (1, 3). Many of these foods are characterized by high densities of salt, added sugar, and fats, and when consumed in high amounts, they can undermine diet quality. In their guiding principles for sustainable and healthy diets, FAO in 2019 for the first time included the processing dimension in their advice, in Principle 2, stating that sustainable and healthy diets ‘… are based on a great variety of unprocessed or minimally processed foods, balanced across food groups, while restricting highly processed food and drink products’ (4).

Food processing is not a recent invention and *per se* not a public health concern and may confer many benefits. Humans have used heat, fermentation, drying, and other processes to avoid spoilage, increase palatability, remove toxins, and ensure microbiological safety of foods since ancient times (5). However, advances in food science and food technology in recent decades to novel processing techniques and food ingredients have allowed for the creation of a range of new and highly processed foods and drink products (6, 7). While an official definition of UPFs is lacking, the vast majority of research works define UPF according to the NOVA framework (8). While there is longstanding and ample evidence showing an association between specific foods (e.g. sugar-sweetened beverages) and nutrients (e.g. sodium and trans fats) and increased risk of chronic diseases like type 2 diabetes and coronary heart disease (9), studies on UPFs are fairly recent.

NOVA classifies foods into four mutually exclusive groups based on the extent and purpose of the industrial processing they have undergone: (1) ‘unprocessed or minimally processed foods’, including fresh, dry, or frozen fruits or vegetables, grains, legumes, meat, fish, and milk; (2) ‘processed culinary ingredients’, including table sugar, oils, fats, salt, and other constituents extracted from foods or from nature and used in kitchens to make culinary preparations; (3) ‘processed foods’, including foods such as canned fish and vegetables, simple breads, and cheeses, which are manufactured by only adding salt, sugar, oil, or other processed culinary ingredients to unprocessed or minimally processed foods; and (4) ‘ultra-processed foods’, which are formulations of ingredients, mostly exclusively of industrial use, that result from a series of industrial processes (8). As a result, UPFs usually contain little whole foods. In contrast to processed foods, the production of UPFs involves a number of novel processing techniques (e.g. extrusion and molding), ingredients (e.g. modified starches and protein isolates), and additives (e.g. emulsifiers and artificial flavors) (8). Examples of UPFs include soft drinks, salty snack foods, fast foods, and candy (8). Many foods that are marketed and perceived as healthy, such as reduced-calorie/low-fat products, are categorized as ultra-processed (8). Foods such as industrially produced breads, breakfast cereals, and flavored yogurts are classified as processed or UPFs depending on their ingredients (e.g. content of cosmetic food additives) (8). More detailed examples of foods included in each NOVA group are available elsewhere (1, 10). The purpose of the NOVA framework is to classify foods according to the extent and purpose of processing it has been submitted to, and the classification does not consider the nutrient composition of foods.

Food processing level has emerged as a novel dimension of diet quality, and UPFs are increasingly scrutinized as a potential driver of the current global epidemics of diet-related chronic diseases (1, 11). Epidemiological studies have consistently found that diets with a higher proportion of UPFs have less favorable nutrient profiles than diets containing less UPFs (10, 12–19). Specifically, diets higher in UPFs are generally higher in total energy, total fat, saturated fat, trans fat, added/free sugars, and sodium while providing less protein, fiber, and several essential vitamins and minerals, although nutrients may be added (1). Furthermore, greater intakes of UPFs have been linked to increased risk of several chronic diseases, including risk of obesity, diabetes, hypertension, dyslipidemia, cardiovascular diseases (CVDs), and all-cause mortality in several large cohort studies (1, 3). In response to the current evidence, some countries have recently implemented public health policies to decrease the consumption of UPFs. For example, Brazil (20), Israel (21), Peru (22), Belgium (23), Ecuador (24), and Uruguay (25) have developed FBDGs dissuading UPF consumption; and Chile has implemented strict food-marketing and front-of-package labeling legislation for unhealthy packaged foods and drinks (26).

Nevertheless, the role of food processing level relative to traditional nutrient-focused metrics in relation to health is under ongoing scientific debate. In particular, the NOVA framework has been criticized as ambiguous and inconsistent, and some argue that the UPF group is too broad and heterogeneous to draw meaningful conclusions regarding its association to health outcomes (27–29). Some scholars also question the usefulness of focusing on processing level beyond conventional nutrient-centric classification systems, arguing that ultra-processed diets are detrimental to health simply because they are of poor nutritional quality (27, 28). Indeed, UPF intake tends to be inversely correlated with diet quality measured by nutrient profile indices, such as the Healthy Eating Index (30), the Nutri-Score (31), and the Nutrient Rich Food Index (32). However, some UPFs are identified as ‘healthy’ based on nutrient profiling (31). As a result, some reason that avoidance of UPFs may negatively impact nutrient intakes (29). On the other hand, if UPFs influence health through non-nutrient-mediated pathways, it is questionable if a food can be considered ‘healthy’ or ‘unhealthy’ solely based on its nutrient composition and if nutrient reformulations are sufficient to address the issues surrounding UPFs (31).

The objective of this scoping review is to evaluate the totality of the available empirical evidence regarding UPFs in relation to health-related outcomes as a basis for setting FBDGs for the Nordic Nutrition Recommendations 2023 (NNR2023) (Box 1). An evaluation of the health effects of food processing in general is beyond the scope of the scoping review and will not be discussed.

*Box 1.* Background papers for Nordic Nutrition Recommendations 2023

- This paper is one of many scoping reviews commissioned as part of the Nordic Nutrition Recommendations 2023 (NNR2023) project (33)
- The papers are included in the extended NNR2023 report, but, for transparency, these scoping reviews are also published in Food & Nutrition Research
- The scoping reviews have been peer reviewed by independent experts in the research field according to the standard procedures of the journal
- The scoping reviews have also been subjected to public consultations (see report to be published by the NNR2023 project)
- The NNR2023 committee has served as the editorial board
- While these papers are a main fundament, the NNR2023 committee has the sole responsibility for setting dietary reference values in the NNR2023 project

## Methods

The current review of the available empirical evidence related to UPFs and non-communicable diseases was conducted in accordance with the protocol developed within the NNR2023 (33, 34). All sources of evidence considered in this chapter adhere to the eligibility criteria determined by the NNR2023 project (33, 34).

The Population, Intervention (or exposure), Comparator, Outcome(s), Timing, Setting, Study design (PI/ECOTSS) statement defining the review topic is presented in Table 1. The NNR2023 project conducted an initial scoping review. Given that the current topic of UPFs is a rapidly growing area of research, the authors conducted additional systematic literature searches on PubMed (MEDLINE) to identify recently published high-quality systematic reviews, meta-analyses, randomized controlled trials (RCTs), and prospective cohort studies examining the association between UPF intake and non-communicable diseases or mortality. Cross-sectional and ecological studies, narrative reviews, case studies, articles in non-English language, and articles not adequately describing research methods or presenting multivariable adjusted risk estimates were excluded. The main literature search was performed on April 12th, 2021. An updated literature search was performed on February 27th, 2022.

**Table 1:** PI/ECOTSS statement defining the review topic of the current chapter

Population	Intervention or exposure	Comparators	Outcomes	Timing	Setting	Study design
All groups: Pregnant women Children Adolescents Adults	Degree of ultra-processed foods in the diet	No/low intake vs high intake of UPFs	Non-communicable diseases Mortality	Published in 2011–2022	General population	Prospective cohort studies, RCTs, Meta-analyses, Systematic reviews

PI/ECOTSS: Population, Intervention (or exposure), Comparator, Outcome(s), Timing, Setting, Study design; UPF: ultra-processed foods; RCT: randomized controlled trials.

**Fig. 1:**
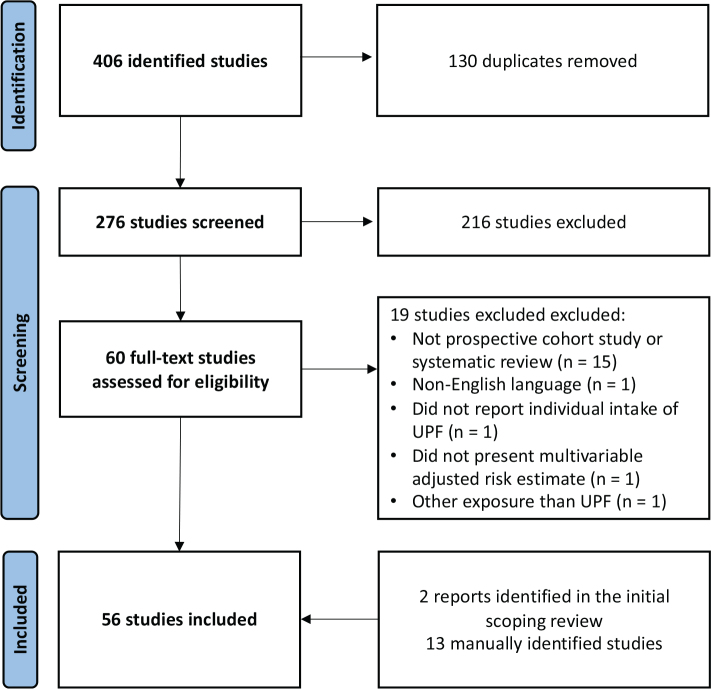
PRISMA flowchart of the systematic search and screening process.

After removal of duplicates, the literature searches yielded a total of 276 unique publications. All titles, abstracts, and full text-articles were screened independently by the two authors. Conflicts were resolved by discussion. A total of 216 articles were excluded based on the title and abstract as they did not meet the inclusion criteria outlined earlier. The authors assessed the remaining 60 full-text articles, of which 19 records did not meet the inclusion criteria and were excluded (see flowchart in Figure 1 for details). Fifteen additional relevant publications were identified by manually searching the bibliographies of the included studies and through the peer-review process of the chapter draft. In total, the current review included 56 articles (2 reports (1, 11), 10 systematic reviews [including 5 meta-analyses] (3, 35–43), and 44 original research studies) (44–86). As instructed by the NNR2023 project, the authors quality-checked all major publications which were directly relevant for setting FDBGs using RoB 2.0 for RCTs and Rob-NObS for observational studies (87, 88).

## Dietary intake in Nordic and Baltic countries

Only a few studies in the peer-review literature provide consumption estimates of UPFs in the Nordic and Baltic countries. Using food consumption data collected by food records or 24-h dietary recalls between 2005 and 2014, Mertens et al. estimated that UPFs accounted for 25.3 and 24.7 %kcal among Danish men and women, 17.4 and 18.4 %kcal among Estonian men and women, 32.0 and 37.3 %kcal among Latvian men and women, and 40.6 and 43.8 %kcal among Swedish men and women (89). Data from household budget surveys in 19 European countries collected between years 1998 and 2008 indicate that UPFs accounted for 26% of the diet by weight, on average (90). Among the Nordic and Baltic countries included in the study, the average proportion of UPFs in the diet (total household food availability in kcal/person per day) was 26% in Lithuania, 33% in Latvia, 37% in Norway, and 41% in Finland (90). In an analysis of food frequency questionnaire data, Borge et al. found that UPFs provided on average 32% of the total energy consumption in a sample of almost 78,000 pregnant women in the Norwegian Mother, Father and Child Cohort Study (64). An analysis of sales data from food retailers reported that UPFs represented 59% of the number of purchased items and 49% of food expenditure in Norway in 2013 (91). In Sweden, the annual per capita consumption of UPFs increased from 125 kg in 1960 (20% of total diet by weight) to 302 kg in 2010 (38% of total diet by weight) (92).

Details regarding the consumption of specific UPFs and foods that may be ultra-processed depending on their ingredients can be found in other parts of the NNR2023 report: sugar-sweetened beverages, artificially sweetened beverages, processed meat, processed fish, margarine, breakfast cereals, bread, savory snacks, sweets, yogurts, ready-to-eat meals, and fast food.

## Health outcomes relevant for Nordic and Baltic countries

### Adults

#### All-cause mortality

A total of six high-quality prospective cohort studies conducted in the United States (49, 50), Spain (58, 59), France (60), and Italy (52), with sample sizes ranging from 3,003 to 22,810 adults, evaluated all-cause mortality in relation to UPF intake (Supplementary Table 1). Greater consumption of UPFs was associated with higher risk of all-cause mortality in five studies (50, 52, 58–60), while one study (49) reported a null association.

Three separate meta-analyses (Supplementary Table 2) found that high vs low intake of UPFs was associated with an increased risk of all-cause mortality (RR: 1.25, 95% CI: 1.14, 1.37 (3); HR: 1.28, 95% CI: 1.11, 1.48 (40); and HR: 1.21, 95% CI: 1.13, 1.30 (42), respectively).

#### Obesity

One American RCT (44) and seven high-quality prospective cohort studies conducted in Spain (45, 46), France (47), the UK (67), Brazil (48), China (66), and the multinational European Prospective Investigation into Cancer and Nutrition (EPIC) study (93) examined the association between UPF intake and weight gain or excess adiposity (Supplementary Table 3).

In an in-patient randomized cross-over trial by the US National Institute of Health, participants (*N* = 20) gained on average 0.9 ± 0.3 kg, primarily in fat mass, when receiving an ad libitum ultra-processed diet (83% energy from UPFs) for 14 consecutive days (44). In contrast, participants lost 0.9 ± 0.3 kg when receiving an ad libitum minimally processed diet for 14 days. The two diets were matched for presented calories, macronutrients, sugar, fiber, and overall energy density; however, the diets differed in the proportion of added vs. naturally occurring (intrinsic) sugar and fiber, and in non-beverage energy density. The findings support that a diet high in UPF increases energy intake and promotes weight gain in the short-term.

Prospective analyses in the EPIC cohort (*n* = 348,748), the NutriNet-Santé cohort (*N* = 110,260), the Seguimiento de Navarra study (SUN; *N* = 8,451), the Brazilian Longitudinal Study of Adult Health (ELSA-Brasil; *N* = 4,527), and the PREDIMED-Plus cohort (*N* = 1,485) demonstrated an association between UPF intake and risk of incident overweight/obesity (46, 48, 93), incident obesity (47, 48, 93), weight gain (93), and greater age-related visceral and overall adiposity accumulation (45).

Meta-analytic pooling of the results from the SUN and ELSA-Brasil studies demonstrated a 23% greater risk of overweight/obesity in the highest vs. lowest consumption quartile (RR: 1.23, 95% CI: 1.11, 1.36) (Supplementary Table 2) (3).

#### Cardiovascular disease

Six high-quality prospective cohort studies conducted in the United States (49, 50, 68, 69), France (51), and Italy (52) with sample sizes ranging from 3,003 to 105,159 adults assessed the association between UPFs and CVD incidence (*N* = 3) and/or mortality (*N* = 4) (Supplementary Table 4). A dose–response association was observed in all studies assessing CVD incidence (49, 51, 68) and three out of the four studies evaluating CVD mortality (49, 52, 69).

Meta-analytic pooling indicated that the highest intake level of UPF was significantly associated with a 29% increased risk of CVD incidence and 34% increased risk of cerebrovascular disease incidence (Supplementary Table 2) (3). Two meta-analyses reported an increased risk for CVD mortality (RR: 1.29, 95% CI: 1.12, 1.48 (3) and HR: 1.50, 95% CI: 1.37–1.63 (42), respectively), cerebrovascular disease mortality (RR: 1.34, 95% CI: 1.07, 1.68) (3), and heart disease mortality (HR: 1.66, 95% CI: 1.50–1.85) (42).

#### Type 2 diabetes

The association between UPF intake in relation to incident type 2 diabetes was investigated in four prospective cohort studies, including the NutriNet-Santé cohort (*N* = 104,707; mean follow-up 6.0 years) (53), the UK Biobank cohort (*n* = 21,730; median follow-up 5.4 years) (73), the Lifelines cohort study (*n* = 70,421; median follow-up 3.4 years) (72), and the SUN study (*n* = 10,060; median follow-up 12 years) (74). Higher intake of UPFs was associated a greater risk of developing type 2 diabetes in all studies (Supplementary Table 4).

A meta-analysis of the four prospective cohort studies (as well as one cross-sectional study) observed a linear dose–response association between UPF intake and diabetes risk, such that each 10% increase in UPF intake (kcal/day) was associated with a 15% higher risk of type 2 diabetes (RR: 1.15, 95%CI 1.36–2.22; *I*^2^ = 86.0%; *p* < 0.001) (Supplementary Table 2) (41).

#### Other cardiometabolic conditions

UPF intake was associated with greater risk of incident hypertension in the SUN cohort (*N* = 14,790; median follow-up 9.1 years; HR: 1.21, 95% CI: 1.06, 1.37 for tertile 3 vs.1; P for trend = 0.004), but not in the Mexican Teachers’ Cohort (*N* = 64,934; median follow-up 2.2 years; IR: 0.98, 95% CI: 0.84, 1.14 for >45 vs. ≤20%kcal from UPF, p-trend: 0.57) (71). An association was initially observed in the ELSA-Brasil study (*N* = 8,754; mean follow-up 4 years); however, the association did not remain significant after adjustment for BMI (54). In the Seniors-Study on Nutrition and Cardiovascular Risk in Spain (ENRICA) cohort (*n* = 1,082; 5–7 years of follow-up), UPF intake was associated with incident hypertriglyceridemia and low high-density lipoprotein (HDL) cholesterol, but not with high low-density lipoprotein (LDL) cholesterol (75). Study details are shown in Supplementary Table 4.

#### Cancer

The association between UPF intake and incident cancer has, to the authors’ knowledge, only been evaluated prospectively in one study. In the NutriNet-Santé cohort (*N* = 104,980; mean follow-up 5.0 years), UPF intake was associated with higher risk of overall cancer (HR for a 10% absolute increment in UPF proportion: 1.12, 95% CI: 1.06, 1.18) and breast cancer (HR: 1.11, 95% CI: 1.02, 1.22), but not of prostate cancer or colorectal cancer (Supplementary Table 5) (55). Additional adjustment for dietary intake of fat, sodium, and carbohydrates did not alter the significance of the observed associations. UPF consumption was not associated with cancer mortality in the SUN study (58) or in the Moli-San study (52).

#### Depression

A greater intake of UPFs was associated with higher risk of incident depression in two high-quality prospective cohort studies conducted in Spain (*N* = 14,907) (56) and France (*N* = 26,730) (57), with a mean follow-up of 10.3 and 5.4 years, respectively (Supplementary Table 5).

Meta-analytic pooling of the two studies indicated a significant association between the intake of UPF and depression (RR: 1.20, 95% CI: 1.03, 1.40 for quartile 4 vs. 1 (3), and HR: 1.22; 95% CI: 1.16, 1.28; Supplementary Table 2) (40).

#### Other health outcomes

UPF intake was associated with higher risk of incident frailty (4th vs. 1st quartile, OR: 3.67, 95% CI: 2.00, 6.73) in the Spanish Seniors-ENRICA Cohort Study (*N* = 1,822 adults aged >59 years, mean follow-up: 3.5 years) (61). Another analysis in the same cohort found that UPF consumption was associated with renal function decline (*N* = 1,312; OR: 1.74, 95% CI: 1.14–2.66 for the highest vs lowest consumption tertile) (78). UPF intake was also associated with hyperuricemia in the Tianjin Chronic Low-grade Systemic Inflammation and Health cohort study in China (*N* = 18,444; mean follow-up 4.2 years). Compared to lowest quartile, the highest quartile had a HR of 1.16 (95% CI: 1.05, 1.28) (77). UPF consumption was associated with incident inflammatory bowel disease in the PURE cohort study (*N* = 116,087; median follow-up 9.7 years; HR: 1.82, 95% CI: 1.22, 2.72 for ≥5 servings/day compared to <1 serving/day) (76), but not in the NutriNet-Santé cohort (*N* = 105,832; mean follow-up 2.3 years) (62). Study details are shown in Supplementary Table 5.

### Pregnancy

Evidence regarding the impact of UPF in pregnancy is limited and currently only addressed by four prospective cohort studies. Greater maternal intake of UPFs during pregnancy was associated with greater gestational weight gain and adiposity of the neonate among a small sample of US women (*N* = 45) (63). Likewise, UPF intake in the third, but not the second, trimester was associated with greater gestational weight gain in a sample of 259 Brazilian women (79). Pre-pregnancy UPF intake was not associated with gestational diabetes in the Spanish SUN study (*n* = 3,730) (80). However, a significant association was observed among women >30 years in age-stratified analyses. In the Norwegian Mother, Father and Child Cohort Study, greater maternal intake of UPFs during pregnancy was associated with increased attention deficit/hyperactivity disorder (ADHD) symptoms in the offspring (*N* = 37,787), but not with the child’s relative risk of ADHD diagnosis at age 8 (*N* = 77,768) (64). Study details are shown in Supplementary Table 6.

### Children and adolescents

A total of seven prospective cohort studies examining UPF consumption in relation to chronic disease outcomes in children were identified (Supplementary Table 7) (65, 81–86). Three of the studies were based on the same small Brazilian cohort of 3–4 year-old children of low socioeconomic status (*N* = 345) (83, 84, 86). In this cohort, greater intake of UPF at age 3 years was associated with higher levels of total cholesterol and triglycerides at age 6 years, higher increase in total cholesterol and LDL cholesterol from age 3–4 to 7–8 years, and greater waist circumference at age 8 years (83, 84, 86). No association was observed between UPF intake at 3 years of age and BMI, waist to height ratio, sum of skinfolds, glucose, insulin or HOMA-IR, HDL-cholesterol, non-HDL cholesterol, and triglycerides at ages 6–8 years (83, 84, 86).

Two studies analyzed data from the Brazilian 2004 Pelotas Birth Cohort Study (65, 82). In an analysis of 3,454 children, each 100g/day increment of UPFs at age 6 years was associated with a 0.14 kg/m^2^ increase in fat-mass index at age 11 years (82). There was no association between UPF intake at 6 years and wheeze, asthma, or severe asthma at 11 years among a smaller subset of the same cohort (*N* = 2,190) (65).

In the UK Avon Longitudinal Study of Parents and Children (ALSPAC) cohort (*n* = 9,025; median follow-up 10.2 years), greater UPF intake at age 7–13 years was associated with a higher weight, BMI, fat-mass index, and waist circumference in young adulthood. No association was observed for lean mass index (81). UPF intake at age 4 years (β = 0.028; 95% CI 0.006, 0.051), but not at age 7 years, was significantly associated with BMI *z*-score at age 10 years in the Portuguese Generation XXI cohort (*n* = 1,175) (85).

## Mechanisms

Processing may alter a food’s health potential by removing or adding macro- and micronutrients, removing naturally occurring bioactive components, altering bioavailability of nutrients, introducing food additives and substances formed during processing (e.g. acrylamide), and modifying the physical structure of the food matrix (94, 95). The biological pathways through which UPFs may influence chronic disease outcomes, such as obesity, cancer, hypertension, type 2 diabetes, CVD, and depression, have not yet been determined, but the current evidence suggests several hypothesized mechanisms.

First, UPFs may contribute to chronic diseases through their poor nutritional profile and by displacing nutritious and health-promoting minimally processed foods, such as fruits, vegetables, whole grains, meat, and fish from the diet (1, 96). However, the available literature suggests that diet quality does not explain the observed associations between diets high in UPFs and health outcomes. A recent review evaluated the relative impact of dietary adjustment on the association between UPF intake and health outcomes in 37 prospective cohort studies (97). A total of 64 out of 66 models demonstrating a significant association between UPF intake and health outcomes remained significant after adjustment for diet quality or diet pattern (97). Out of 142 dietary adjustments, 136 did not explain the association between UPF intake and the relevant outcome (97).

Second, UPFs are generally more hypercaloric and less satiating than minimally processed foods and may therefore facilitate excessive energy intakes (98). Notably, the RCT by Hall and colleagues demonstrated that an ultra-processed diet (83.5% of total energy from UPFs) increased ad libitum energy intake by ~500 kcal/day compared to a minimally processed diet with a similar nutrient profile (44). The average eating rate, measured as grams/minute and kcal/minute, was significantly higher during the ultra-processed diet compared to the minimally processed diet, which, in combination with the higher non-beverage energy density of the ultra-processed diet, may have contributed to greater overall energy intake (44). Experimental studies support that food texture influences eating rate, and that soft UPFs lead to higher eating rates and ad libitum energy intakes, compared to both minimally processed foods and UPFs with hard textures (99). Furthermore, in the RCT by Hall et al., higher levels of the appetite suppressing hormone Peptide YY were noted during the minimally processed diet compared to the ultra-processed diet, suggesting that processing level may influence energy intake through hormonal pathways (44).

It is also hypothesized that the convenience, omnipresence, affordability, large portion sizes, and persuasive marketing of UPFs promote poor dietary habits, snacking and over-eating, which, in turn, may lead to increased energy intake and weight gain (96). Accordingly, two recent meta-analyses of observational studies found a significant association between consumption of UPFs and overweight (3, 35).

Third, processing can alter the physical structure of the food matrix, with potential implications for nutrient bio-accessibility, absorption kinetics, and the gut microbiome (95, 100, 101). The large share of acellular nutrients (macronutrients that have been rendered completely devoid of any natural intact food structure) in UPFs and consequent high nutrient availability in the small intestine may promote an inflammatory gut microbiota (100, 101). UPFs are generally low in dietary fiber, which provide substrate for microbial fermentation. Western-style diets that are low in fiber while high in sugar and fat are associated with a distinct and less diverse microbiotic profile compared to diets rich in minimally processed plant foods (102). Rodent studies also indicate that low-fiber diets may shift the gut microbial metabolism toward the utilization of proteins and host mucins, resulting in degradation of the intestinal mucus layer and increased susceptibility to chronic inflammatory diseases (102, 103). In contrast, meta-analyses of human RCTs demonstrate that increased intakes of dietary fiber can significantly improve glycemic control and insulin sensitivity, decrease total cholesterol and LDL cholesterol, and reduce the risk of developing type 2 diabetes (104, 105).

Fourth, additives and other ingredients of exclusive industrial use in UPFs may influence biological systems and health outcomes. For example, experimental studies in both humans and animals indicate that non-nutritive sweeteners (106–108) and certain emulsifiers (carboxymethylcellulose and polysorbate 80) (109, 110) may disrupt gut microbiota integrity and promote a pro-inflammatory status and metabolic dysregulation. In an RCT involving 120 healthy adults, daily consumption during 2 weeks of the non-nutritive sweeteners saccharin, sucralose, aspartame, and stevia in doses lower than the acceptable daily intake each resulted in distinctly altered fecal and oral microbiome and plasma metabolome, with individual differences (108). Intake of saccharin and sucralose also significantly impaired glucose metabolism (108). Another RCT demonstrated that the consumption of sucralose in combination with a carbohydrate impairs insulin sensitivity in healthy individuals, potentially due to dysregulation of the gut-brain regulation of glucose metabolism (107). Long-term consumption of sucralose (10 weeks) also increased serum insulin levels and altered glucose response in healthy adults (111). The frequent use of phosphate salts in industrial food processing may lead to excessive phosphorous intakes, which can disrupt the hormonal regulation of extra-cellular phosphate and promote arterial calcification, cause oxidative stress of the endothelial cells, and impair endothelial function (112). Although food additives must be evaluated for safety, they are not tested for effects on gut microbiota, immune responses, and metabolism prior to approval (113).

Fifth, extensive heat treatment and extruding during processing may lead to the formation of contaminants. For example, advanced glycation-end products have been linked to increased oxidative stress and inflammation (114); acrolein (115) and acrylamide (116) have been linked to insulin resistance, and polycyclic aromatic hydrocarbons have been associated with diabetes (117). Furthermore, industrial partial oil hydrogenation may lead to the creation of trans-fatty acids, which are linked to CVD and diabetes (9, 118).

Finally, limited epidemiologic data support that ultra-processed intake is associated with increased exposure to endocrine-disrupting chemicals and phthalates used in industrial plastic packaging (119). For example, bisphenol A (BPA) has been shown to promote insulin resistance, oxidative stress, inflammation, adipogenesis, and pancreatic beta-cell dysfunction by binding to estrogen-related receptors (120). While bisphenol A is banned for use in food packaging in many countries, it is often replaced by similar components such as bisphenol S, which also has endocrine-disrupting properties (119).

In summary, UPFs may contribute to metabolic disturbances and inflammatory processes, which are present in obesity, cardiometabolic diseases, cancer, and depression.

## Food-based dietary guidelines

### Summary of main results

Higher consumption of UPFs was consistently associated with increased risk of weight gain, obesity, CVD, and type 2 diabetes in prospective cohort studies. While there were some inconsistent findings, most studies also reported an association between UPF intake and all-cause mortality. The strongest evidence is observed in relation to weight gain, as this association is supported by both epidemiological studies and an RCT. In the case of hypertension, cancer, and depression, the limited number of studies and subjects investigated preclude strong conclusions.

All the included cohort studies concerning weight gain, obesity, CVD, type 2 diabetes, and all-cause mortality had large sample sizes, adequate follow-up time, and high participation rates, which strengthens the current evidence base. Using the Risk of Bias for Nutrition Observational Studies (RoB-Nobs) Tool (88), the risk of bias due to confounding in these studies was determined as ‘low’ to ‘moderate’ for all domains. Risk of bias assessment due to other domains such as selection of study participants, classification of exposures, departures from intended exposure, missing data, measurements of outcomes, and selection of reported results were not performed in the present scoping review. Although the RCT by Hall et al. was not blinded, the risk of bias in this study was deemed as ‘low’, using the revised Cochrane risk of bias tool for randomized trials (RoB 2.0) for crossover trials (87), indicating good methodological quality.

Overall, a key finding of this review is that the observed associations largely remained significant despite adjustment for nutrient intakes and indicators of diet quality or patterns, suggesting that the nutritional composition of UPFs alone does not explain the excess disease risk associated with their consumption.

It should be noted that a shared limitation of most of the included cohort studies is the lack of dietary assessment methods and nutrient databases specifically designed to assess UPF intake, which may have led to misclassification of individual foods and measurement error. Under- and overestimation of UPF intake due to misclassification of food items may have attenuated or strengthened the observed associations. However, misclassification error would likely be random, which would bias the associations toward the null. Future studies should use dietary assessment tools that have been validated for collecting data regarding UPF intake. Furthermore, nutrient databases should be enhanced with brand-specific data to better distinguish between similar foods of differing processing levels.

### Data gaps for future research

Additional well-conducted cohort studies in diverse populations and settings are needed, particularly in relation to hypertension, cancer, and depression in adults. Investigations in children and pregnant women are lacking for all health outcomes and should be prioritized. Given that there are national differences in food supplies, health status, and culinary traditions, more studies in Nordic and Baltic populations are warranted. Nevertheless, the reviewed studies were conducted in multiple different countries and populations, including various European countries, which increases the generalizability of the results. Where ethically feasible, experimental studies should be conducted to examine potential causal association between UPF intake and health outcomes, using biomarkers or intermediary outcomes (e.g. blood pressure and blood lipids).

Further research is also warranted to clarify the biological mechanisms through which UPFs may influence health outcomes and the proportional harm associated with the nutritional composition, food additives, physical structure, and other properties of UPFs. One crucial question is whether diets based on UPF promote passive overconsumption in the long-term beyond effects explained by traditional dietary risk factors (27). While the RCT by Hall et al. suggests this is the case in the short-term (44), long-term studies are needed to clarify this controversy. Understanding how ultra-processing changes whole foods and through which pathways these foods affect health is a prerequisite for eliminating harmful processing techniques and ingredients and identifying ‘optimal’ vs. detrimental types of processing. Given that UPFs constitute a large and heterogeneous category, which includes foods that have undergone different processes and differ in ingredients and nutrient profiles, the role of specific exposures (e.g. subgroups of UPFs, additives, etc.) needs to be examined in experimental and/or epidemiological studies to clarify biological mechanisms. The effects of UPFs on the gut microbiota and microbiota–host interactions constitute an area of special scientific interest, given the accumulating evidence regarding the role of the gut microbiome in cardiometabolic health and diet-disease relationships. Finally, research is needed to examine the potential benefits of dietary advice focusing on processing level in addition to nutrient-based recommendations in promoting and maintaining improvements in food choices and diet quality.

### Integration

Although fairly new, the concept of UPFs has already gained wide acceptance among many health researchers. Some scholars, however, have criticized the NOVA framework as ambiguous and questioned its usefulness for informing dietary guidelines beyond conventional nutrient-based classification systems (27). Nonetheless, the available evidence suggests that the adverse health outcomes associated with UPF intake are independent of nutrient content and overall dietary quality and patterns (97). UPFs have also been shown to facilitate excessive energy intakes and promote weight gain in the short-term (44). While the exact underlying mechanisms linking UPFs to chronic diseases are not yet fully elucidated, several factors beyond nutritional composition, such as food additives and physical structure, may play a role (96, 101). Therefore, limiting the intake of UPFs may offer additional advantages to solely limiting consumption of foods that are high in salt, sugar, and fat, or changing the nutritional composition of UPFs through reformulation.

Furthermore, diets high in UPFs tend to be high in foods and nutrients that should be limited according to the current FBDGs, including processed meats, sweets, sugar-sweetened beverages, refined grains, and added sugars, while low in recommended dietary components such as fruit, vegetables, whole grains, and fiber (1). Evidence also suggests that UPFs are, on average, more energy-dense (2.2 vs. 1.1 in kcal/g) and nutrient-poor (Nutrient Rich Food index per 100 kcal: 21.2 vs. 108.5) than minimally processed foods (32). As a result, recommendations to limit UPFs, and choose non-UPFs, when possible, may enhance and support several of the existing FBDGs and help individuals select more healthful foods that align with the overall NNR2023 guidelines within each food category.

## Conflict of interest and funding

The authors received a small reimbursement from the Norwegian Directorate of Health for the work associated with this scoping review. The authors have no conflicts of interest.

## Supplementary Material














