# Supplementary material for: Ultra-processed foods – a scoping review for Nordic Nutrition Recommendations 2023
Source: Food Nutr Res. 2024 Apr 24;68:10.29219/fnr.v68.10616. doi: 10.29219/fnr.v68.10616 (PMC11077402; doi:10.29219/fnr.v68.10616)
Supplement: Supplementary file 1 [file FNR-68-10616-s1.docx]

| Reference | Study design | Population | Dietary assessment | Exposure | Outcomes | Main results | Covariates |
| --- | --- | --- | --- | --- | --- | --- | --- |
| *Juul et al. 2021* | Prospective cohort study  Mean follow-up 20.0 years | The Framingham Offspring Cohort, United States  Adults >25 years  N=3003 | FFQ  UPF defined by NOVA | UPF (energy adjusted servings/day) | All-cause mortality | No association  (HR: 1.01, CI: 0.99, 1.04) | Age, sex, education,  smoking status, alcohol intake, physical activity. |
| *Blanco-Rojo et al. 2019* | Prospective cohort study  Mean follow-up 7.7 years | The ENRICA Cohort, Spain  Representative sample of non-institutionalized adult population  N=11,898 (mean age 46.9 years, 50.5% women) | FFQ, dietary history  UPF defined by NOVA | UPF (% of energy, divided in quartiles) | All-cause mortality | Greater UPF intake associated with greater all-cause mortality risk (4^th^ vs 1^st^ quartile, HR: 1.44, CI: 1.01, 2.07; P trend = 0.03) | Age and sex, education, living alone, smoking,  former drinker, physical activity, time watching television, time devoted to other sedentary activities,  number of medications per day and presence of chronic  conditions. |
| *Kim et al. 2019* | Prospective cohort study:  Median follow-up of 19 years | The Third National Health and Nutrition Examination Survey (NHANES III), United States  Representative sample of non-institutionalized adult population  N=11,898 | FFQ  UPF defined by NOVA | UPF (frequency of intake, divided in quartiles) | All-cause mortality | Greater UPF intake associated with greater all-cause mortality risk (4^th^ vs 1^st^ quartile, HR: 1.30; 95%CI: 1.08, 1.57; P-trend = 0.001). | Age, sex, race/ethnicity, total energy intake, poverty level, education, smoking, physical activity, alcohol intake, hypertension, total cholesterol, estimated glomerular filtration rate. |
| *Rico-Campà et al. 2019* | Prospective Cohort Study  Mean follow-up of 9.1 years | The Seguimiento Universidad de Navarra study, Spain  University graduates free of hypertension at baseline  N=19,899 | FFQ  UPF defined by NOVA | UPF (energy adjusted servings/day, divided in quartiles) | All-cause mortality | Each additional serving of UPF was associated with an 18% increased risk of all-cause mortality (HR: 1.18, 95%CI: 1.05 to 1.33).  Intake >4 servings/day associated with 62% relatively increased hazard for all-cause mortality (HR: 1.62, 95%CI:1.13, 2.33; p-trend=0.005) | Age, sex, marital status, baseline BMI, total energy intake, smoking, family history of cardiovascular  disease, alcohol intake, educational, snacking, following a special diet at baseline, physical activity, lifelong cumulative smoking and baseline presence of cardiovascular disease, cancer, diabetes, hypertension, hypercholesterolemia and depression. |
| *Bonaccio et al. 2021* | Prospective Cohort Study  Median follow-up: 8.2 years | Moli-sani Study, Italy  N=22,475 | FFQ  UPF defined by NOVA | UPF (%g/day, divided into quartiles) | All-cause mortality  Cancer mortality | Greater UPF intake associated with increased risk of  all-cause mortality (4^th^ vs 1^st^ quartile, HR: 1.26; 95%CI: 1.09, 1.46).  Biomarkers of renal function accounted for 20% of the association.  UPF not associated with cancer mortality | Sex, age, energy intake, educational level, housing tenure, smoking, leisure-time physical activity, BMI, history of cancer, CVD, diabetes, hypertension,  hyperlipidemia, residence, and Mediterranean Diet Score |
| *Schnabel et al 2019* | Prospective Cohort Study  Median follow-up: 7.1 years | NutriNet-Santé Study, France  N=44,551 | Repeated 24h-recall  UPF defined by NOVA | UPF (% grams per day) | All-cause mortality | Greater UPF intake associated with a higher risk of all-cause mortality (HR per 10% increment: 1.14; 95%CI: 1.04-1.27; P = 0.008) | Sex, age, household income, educational level, marital status, residence, BMI, physical activity level, smoking status, energy intake, alcohol intake, season of food records, first-degree family history of cancer or cardiovascular diseases, and number of food records and adherence to the French nutritional recommendations |

BMI, Body Mass Index; HR, Hazard ratio; RR, Relative risk; UPF, Ultra-processed foods; WC, Waist circumference, 95%CI, 95% confidence interval
