# Supplementary material for: Ultra-processed foods – a scoping review for Nordic Nutrition Recommendations 2023
Source: Food Nutr Res. 2024 Apr 24;68:10.29219/fnr.v68.10616. doi: 10.29219/fnr.v68.10616 (PMC11077402; doi:10.29219/fnr.v68.10616)
Supplement: Supplementary file 2 [file FNR-68-10616-s2.docx]

**Systematic reviews and meta-analyses**

| Reference | Studies & study design | Population | Exposure | Outcomes | Main results |
| --- | --- | --- | --- | --- | --- |
| ADULTS / ALL AGES | | | | | |
| *Monteiro et al. 2019* | N=22, of which:   - 11 prospective cohort studies - 8 cross-sectional studies - 2 ecological study - 1 RCT | All ages | UPF Intake | - Non-communicable diseases | ***Systematic review***  Most studies showed significant dose-response associations between the dietary share of UPF and the occurrence or incidence of several non-communicable diseases, including obesity and obesity-related outcomes, cardiovascular and metabolic diseases, breast and all cancers, depression,  gastrointestinal disorders, frailty in the elderly, and premature mortality. |
| *dos Santos et al. 2019* | N=11, of which:   - 2 prospective cohort studies - 9 cross-sectional studies | Adults and elderly | UPF intake | - Cardiometabolic factors | ***Systematic review***  UPF was positively associated with overweight and obesity, high blood pressure and metabolic syndrome. |
| *Askari et al. 2020* | N=14, of which:   - 1 prospective cohort studies - 13 cross-sectional studies | All | UPF intake | - Overweight - Obesity | ***Meta-analysis***   - Overweight (OR: 1.02; 95%CI 1.01-1.03) - Obesity (OR: 1.26; 95%CI 1.13-1.41)   ***Systematic review***  Positive association between UPF and excess body weight |
| *Meneguelli et al. 2020* | N=21, of which:   - 6 prospective cohort studies - 13 cross-sectional studies - 2 ecological studies | All ages  Brazil (n=10), Spain (n=2), USA (n=2), Guatemala (n=1), UK (n=1), Norway (n=1), Lebanon (n=1), Canada (n=1), Sweden (n=1) | UPF intake | - Body weight - Metabolic syndrome - Hypertension - Lipid profile | ***Systematic review***  Studies shown a positive association of UPF with excess body weight, hypertension, dyslipidemia and metabolic syndrome. |
| *Chen et al. 2020* | N=20, of which:   - 12 prospective cohort studies - 8 cross-sectional studies | All ages | UPF intake | - Mortality (n=4), including cardiovascular mortality (n=1) - Cardiovascular disease and risk factors including weight (n=11), weight (n=6) - Depression (n=2) - Asthma (n=1) - Cancer (n=1) - Gastrointestinal disorders (n=1) - Frailty (n=1) | ***Systematic review***  UPF consumption was associated withal-cause mortality, overall cardiovascular diseases, coronary heart disease, cerebrovascular diseases, hypertension. Metabolic syndrome, overweight and obesity, depression, irritable bowel syndrome, overall cancer, postmenopausal breast cancer, gestational obesity, adolescent asthma and wheezing, and frailty.  No significant association with cardiovascular disease mortality, prostate and colorectal cancers, gestational diabetes mellitus and gestational overweight. |
| *Lane et al. 2020* | N=43, of which:   - 19 prospective cohort studies - 21 cross-sectional studies - 2 case-control studies - 1 prospective + cross-sectional analysis | All ages  Brazil (n=17), France (n=8), Spain (n=7), Canada  (n=3), United States of America (n=3), United Kingdom (n=2),  Norway (n=1), Lebanon (n=1) and Malaysia (n=1 | UPF intake | - Overweight and   obesity among adults (n=14)   - Other measures of overweight and   obesity, including categorical variables of weight gain and waist circumference or abdominal obesity (n=4)   - overweight and obesity among adolescents (n=4) - overweight and obesity in children (n=2) - All-cause mortality in adults (n=4) - Metabolic syndrome (n=4) - Type 2 diabetes (n=1) - Depression (n=2) - Respiratory illness (n=2) | ***Meta-analysis***  **Adults:** UPF associated with increased risk of   - overweight (OR: 1.36; 95% CI, 1.23-1.51; P < 0.001) - obesity (OR: 1.51; 95% CI, 1.34-1.70; P < 0.001) - abdominal obesity (OR: 1.49; 95% CI, 1.34-1.66; P < 0.0001) - all-cause mortality (HR: 1.28; 95% CI, 1.11-1.48; P = 0.001) - metabolic syndrome (OR: 1.81; 95% CI, 1.12-2.93; P = 0.015) - depression (HR: 1.22; 95% CI, 1.16-1.28, P < 0.001)   **Adolescents:** UPF associated with wheezing (OR: 1.40; 95% CI, 1.27-1.55; P < 0.001) but not asthma (OR: 1.20; 95% CI, 0.99-1.46; P = 0.065).  ***Systematic review***   - UPF associated with: cardiometabolic diseases, frailty, irritable bowel syndrome, functional dyspepsia and cancer (breast and overall) in adults - Metabolic syndrome in adolescents - Dyslipidemia in children |
| *Pagliai et al. 2021* | N=23   - 13 cohort studies - 10 cross-sectional studies |  | UPF intake | **Cross-sectional studies:**   - Overweight/obesity (n=5) - Waist circumference (n=4) - Metabolic syndrome (n=2) - Low HDL (n=3) - Hypertension (n=3) - Hyperglycemia (n=3) - Hypertriglyceridemia (n=3) - IBS (n=1) - CRP (n=1)   **Prospective cohort studies:**   - All-cause mortality (n=5) - CVD (n=3) - Cerebrovascular disease (n=2) - Depression (n=2) - Incident frailty (n=1) | ***Meta-analysis***  **Cross-sectional studies:** Highest UPF was associated with risk of overweight/obesity (+39%), high waist circumference (+39%), low HDL-cholesterol levels (+102%), and metabolic syndrome (+79%).  No association: Hypertension, hyperglycemia.  **Prospective cohort studies:**  High UPF associated with increased risk for; all-cause mortality (RR: 1.25, 95% CI 1.14-1.37). CVD (RR: 1.29, 95%CI 1.12-1.48), cerebrovascular disease (1.34, 95%CI 1.07-1.68), and depression (RR: 1.20, 95%CI 1.03-1.40). |
| *Moradi et al. 2021* | N=5   - 4 prospective cohort studies - cross-sectional study | Adults (≥18 years) | UPF intake | - T2DM (n=5) | ***Meta analysis***   - Higher UPF increased risk for T2DM (RR: 1.15, 95%CI 1.36-2.22) |
| *Jardim et al. 2021* | N=38, of which:   - 16 prospective cohort studies - 19 cross-sectional studies - 3 case-control studies | Adults | UPF intake | - obesity (n=17) - hypertension (n=9) - dyslipidemia (n=4) - diabetes (n=5) - cardiovascular disease (n=2) - cancer (n=4) - chronic kidney disease (n=1) - gastrointestinal tract disease (n=2) - depression (n=1) | ***Systematic review***   - Higher UPF consumption was positively associated with obesity and associated with the development of all NCDs, mainly hypertension, diabetes and dyslipidemia. - Only a few studies have demonstrated the protective effect of natural foods and MPF consumption on the occurrence of NCDs |
| *Suksatan et al. 2022* | N=7 prospective cohort studies | Adults | UPF intake | - All-cause mortality (n=6) - CVD mortality (n=4) - Heart-cause mortality (n=2) - Cancer mortality (n=2) | ***Meta analysis***  UPF associated with increased risk of:   - All-cause mortality (HR: 1.21, 95%CI 1.13-1.30) - CVD mortality (HR: 1.50, 95%CI 1.37-1.63) - Heart-cause mortality (HR: 1.66, 95%CI 1.50-1.85)   No association observed for cancer mortality |
| *Shekar & Popkin, 2020* | Policy document, included studies not specified | All ages | UPF intake | - Obesity | Accumulating evidence link the shift from real foods to UPF to overweight/obesity. |
| CHILDREN & ADOLESCENTS | |  |  |  |  |
| *Costa et al. 2018* | N=26, of which:   - 15 prospective cohort studies - 6 cross-sectional studies - 5 intervention studies | Children and adolescents | Intake of:   - Groups of UPF (n=11) - Soft drinks/ sweetened beverages (n=12) - Other specific UPFs (n= 3) | - Body fat | ***Systematic review***  6 of 11 studies found a direct association between groups of UPF and body fat; 5 studies reported a null association |

BMI, Body Mass Index; HR, Hazard ratio; OR, Odds ratio; RR, Relative risk; UPF, Ultra-processed foods; 95%CI, 95% confidence interval
