# Supplementary material for: Ultra-processed foods – a scoping review for Nordic Nutrition Recommendations 2023
Source: Food Nutr Res. 2024 Apr 24;68:10.29219/fnr.v68.10616. doi: 10.29219/fnr.v68.10616 (PMC11077402; doi:10.29219/fnr.v68.10616)
Supplement: Supplementary file 3 [file FNR-68-10616-s3.docx]

| Reference | Study design | Population | Dietary assessment | Exposure | Outcomes | Main results | Covariates |
| --- | --- | --- | --- | --- | --- | --- | --- |
| *Hall et al. 2019* | Randomized controlled trial | Weight-stable healthy volunteers  N=20  United States | Direct observation | Ultra-processed vs. unprocessed diet defined by NOVA | - △ Energy intake (kcal/day) - △ Body weight (kg) - △ Body fat mass (kg) | Ultra-processed diet led to:   - Greater energy intake (508 ± 106 kcal, p=0.0001) - Weight gain (0.9 ± 0.3 kg, p=0.009) - Body fat mass gain (0.4 ± 0.1 kg, p=0.0015) | N/A |
| *Beslay et al. 2020* | Prospective cohort study  Median follow-up 4.1 years | The NutriNet-Santé cohort, France  Adult volunteers from general population (mean age 43.1 at baseline)  N=110,206 | Repeated 24-h recalls  UPF defined by NOVA | UPF (% of weight), continuous (absolute increments of 10%) and divided into sex-specific quintiles | - △ BMI (kg/m^2^) - Incident BMI ≥25 - Incident BMI ≥30 | Higher UPF intake associated with greater increase in BMI and risk of BMI ≥25 and ≥30.  Per 10% increment in UPF:   - β: 0.02, 95%CI: 0.01, 0.02 - Overweight, β:1.11, 95%CI: 1.08, 1.14 - Obesity, β:1.09, 95%CI: 1.05, 1.13   5^th^ vs 1^st^ quintile:   - BMI, β: 0.04, 95%CI: 0.04, 0.05 - Incident BMI ≥25, HR:1.26, 95%CI: 1.18, 1.35 - Incident BMI ≥30, HR: 1.15, 95%CI: 1.04, 1.28 | Age, sex, educational level, smoking status, marital status,  physical activity level, energy intake, alcohol intake, and number of dietary records.  Further models additionally adjusted for:  #1: intakes of sugar, sodium, saturated, and dietary fiber.  #2: healthy and Western dietary pattern.  #3: consumption of fruit, vegetables and sugary drinks. |
| *Konieczna et al. 2021* | Prospective analysis based on RCT  (lifestyle intervention for weight loss)  1 year of follow-up | PREDIMED-Plus, Spain  Men and women aged 55-75 years  with overweight/ obesity and metabolic syndrome  N=1,485 | FFQ  UPF defined by NOVA | UPF (% of weight), continuous and divided into tertiles | Sex-specific z-scores of:   - Visceral fat - Android-to-gynoid fat ratio - Total fat mass   Measured by DXA | A 10% daily increment in UPF intake was associated with greater accumulation of:   - Visceral fat (β: 0.09, 95%CI: 0.05, 0.13) - Android-to-gynoid fat ratio (β: 0.05, 95%CI: 0.00, 0.09) - Total fat (β: 0.09, 95%CI:0.06, 0.13)   3^rd^ vs 1^st^ tertile:   - Visceral fat (β: 0.13, 95%CI: 0.07, 0.19) - Android-to-gynoid fat ratio (β: 0.11, 95%CI:0.05, 0.16) - Total fat (β: 0.15, 95%CI: 0.11, 0.19) | Age, sex, study arm, follow-up time, educational level,  marital status, smoking habits, type 2 diabetes, height, physical activity, sedentary behavior.  *Sensitivity analyses:* #1: intake of sodium, saturated and trans-fat,  alcohol, fiber, glycemic index, fruit and vegetable  consumption, adherence to Mediterranean diet.  #2: number of MetS criteria diagnosed at  inclusion, history of overweight or medically diagnosed prevalence of depression. |
| *Li et al. 2021* | Prospective cohort study | China Nutrition and Health  Survey  Adults aged 20+ (mean age at baseline 43.7)  N=12,451 | 3 day 24-h recall  UPF defined by NOVA | UPF (g/day), divided into 0, 1-19, 20-49, ≥50 g/day. | - Incident BMI ≥ 25 - Central obesity (WC ≥90 cm) | Greater UPF intake (≥50 vs. 0 g/day) associated with greater risk of:   - BMI ≥ 25 (OR:1.45, 95%CI: 1.21–1.74) - Central obesity (OR:1.50. 95%CI: 1.29–1.74) | Age, sex, and energy intake, income, urbanization, education, fat intake,  smoking, alcohol drinking, and physical activity, dietary patterns. |
| *Mendonca et al. 2016* | Prospective cohort study  Median follow-up 8.9 years | The Seguimiento de Navarra Study, Spain  University graduates (mean age 36.7 at baseline)  N=8,451 | Repeated FFQ  UPF defined by NOVA | UPF (servings/day, divided into quartile) | Incident BMI ≥25 | Greater UPF intake associated with greater risk of incident BMI ≥25 (4^th^ vs 1^st^ quartile, HR: 1.26; 95%CI: 1.10, 1.45; P-trend = 0.001) | Sex, age, marital status, educational status, physical activity, television watching, siesta sleep, smoking  status, snacking between meals, following a special diet at baseline, baseline BMI, and intake of fruit and vegetables  *Sensitivity analyses:*  1. Total E  2. Family history of obesity  3. >3kg weight gain in 5y before baseline |
| *Rauber et al. 2021* | Prospective Cohort Study  Median follow-up 5 years | UK Biobank Study  N = 22,659 | 24h-recall  UPF defined by NOVA | UPF (% total energy), divided into quartiles and as a continuous measure (10% increments) | - BMI - WC - %Body fat (%BF) - Incident BMI≥30 | Greater UPF intake associated with greater risk of (4^th^ vs. 1^st^ quartile):   - Incident BMI≥30   (HR:1.79, 95%CI: 1.06, 3.03)   - Abdominal obesity (HR:1.30; 95%CI: 1.14, 1.48) - A ≥ 5% increase in BMI (HR:1.31, 95%CI:1.20, 1.43), WC (HR: 1.35, 95%CI: 1.25, 1.45) and %BF (HR 1.14; 95%CI: 1.03─1.25) | Age, sex, Index of Multiple Deprivation, current smoking  status, physical activity, sleep duration and BMI, WC or BF at baseline (according to outcome).  . |
| *Cordova et al 2021* | Prospective Cohort Study  5-year follow-up | European Prospective Investigation  into Cancer and Nutrition (EPIC) study  N=348,748 | FFQ (country-specific)  UPF defined by NOVA | UPF (g/day), continuous (per SD = 250g/d) and divided into quintiles | - Weight gain - Incident BMI ≥ 25 - Incident BMI ≥ 30 | Greater UPF intake associated with:   - Greater weight gain (0.12 kg/5 years per SD, 95%CI: 0.09, 0.15). - Greater risk of incident BMI ≥ 25 in normal weight participants (RR: 1.15, 95%CI: 1.11, 1.19, for 5^th^ vs 1^st^ quintile) - Greater risk of incident BMI ≥ 30 in participants with incident BMI ≥ 25 and <30 at baseline (RR: 1.16, 95%CI: 1.09, 1.23, for 5^th^ vs 1^st^ quintile) | Age, sex, country/center, BMI at baseline, follow-up time in years, educational level, levels of physical activity, alcohol intake at baseline, smoking status at follow-up,  plausibility of dietary energy reporting, the modified relative Mediterranean diet score |
| *Canhada et al. 2020* | Prospective cohort study | The Brazilian Longitudinal Study of Adult Health (ELSA-Brasil) cohort.  Civil servants of Brazilian public academic institutions,  aged 35–74 years  N=11,827 | FFQ  UPF defined by NOVA | UPF intake (% total energy), divided into quartiles | - Incident BMI ≥ 25 - Incident BMI ≥ 30 among participants with BMI 25-29.9 at baseline - Large weight gain (1·68 kg/year) - Large waist gain (2·42 cm/year) | Greater UPF intake associated with (4^th^ vs 1^st^ quartile):   - Incident BMI ≥ 25 (RR:1.20, 95%CI:1.03, 1.40) - Large weight gain (RR:1.27, 95%CI: 1.07, 1.50) - Large waist gain (RR:1.33, 95%CI: 1.12, 1.58)   UPF intake was not associated with increased risk of incident BMI ≥ 30 among participants with BMI ≥ 25 and <30 at baseline | Age, sex, color/race, center, income, school achievement, smoking, physical activity; for incident BMI ≥ 25/30 and weight gain, plus baseline BMI; for waist gain, plus waist circumference at baseline. |

BMI, Body Mass Index; HR, Hazard ratio; RR, Relative risk; UPF, Ultra-processed foods; WC, Waist circumference, 95%CI, 95% confidence interval
