# Supplementary material for: Ultra-processed foods – a scoping review for Nordic Nutrition Recommendations 2023
Source: Food Nutr Res. 2024 Apr 24;68:10.29219/fnr.v68.10616. doi: 10.29219/fnr.v68.10616 (PMC11077402; doi:10.29219/fnr.v68.10616)
Supplement: Supplementary file 4 [file FNR-68-10616-s4.docx]

| Reference | Study design | Population | Dietary assessment | Exposure | Outcomes | Main results | Covariates |
| --- | --- | --- | --- | --- | --- | --- | --- |
| CVD INCIDENCE & MORTALITY | | | | | | | |
| *Du et al. 2021* | Prospective cohort study  Median follow-up 27 years | Atherosclerosis Risk in Communities study, USA  Aged 45-65  N=13,548 | FFQ  UPF defined by NOVA | UPF (servings/day), divided into quartiles | Incident coronary artery disease (CAD) | Greater UPF intake associated with greater risk of incident CAD (4^th^ vs 1^st^ quartile, HR: 1.19; 95%CI: 1.05, 1.35) | Age, sex, total energy intake, race, study center, education, smoking, drinking status,  physical activity. |
| *Juul et al. 2021* | Prospective cohort study  Mean follow-up 18.0 years (CVD) and  20.0 years (mortality) | The Framingham Offspring Cohort, United States  Adults >25 years  N=3003 | FFQ (NOVA) | UPF (energy adjusted servings/day) | Incident:   - Hard CVD - Hard CHD - Overall CVD   CVD mortality | Each additional daily serving of UPF was associated with:   - 7% increased risk of hard CVD (HR: 1.07, 95%CI: 1.03, 1.12) - 9% increased risk of hard CHD (HR: 1.09, 95%CI: 1.04, 1.15) - 5% increased risk of overall CVD (HR: 1.05, 95%CI: 1.02, 1.08) - 9% increased risk of CVD mortality (1.09, 95%CI: 1.02, 1.16) | Age, sex,  education,  smoking status, alcohol intake, physical activity.  *Sensitivity analyses*: Additionally adjustement for  #1. Total energy intake  #2. Diet quality  #3. Waist circumference  #4. BMI |
| *Srour et al. 2019 A* | Prospective cohort study  Mean follow-up 5.2 years | The NutriNet-Santé cohort, France  Adult volunteers from general population  N=105,159 | Repeated 24-h recalls  UPF defined by NOVA | UPF (% of weight)   - Continuous (increments of 10%) - Quartiles | Incident:   - CVD - CHD - Cerebrovascular disease | Higher UPF intake was associated with increased risk of CVD, CHD and cerebrovascular disease.  Per absolute increment of 10% UPF:   - CVD, HR: 1.12, 95%CI: 1.05, 1.20 - CHD, HR: 1.13, 95%CI: 1.02, 1.24 - Cerebrovascular disease, HR: 1.11, 95%CI: 1.01, 1.21   Quartile 4 vs 1:   - CVD, HR: 1.23, 95%CI: 1.04, 1.45; p-trend=0.02 - CHD, HR: 1.20, 95%CI: 0.93, 1.53; p-trend=0.07 - Cerebrovascular disease, HR:1.24, 95%CI:1.00, 1.53; p-trend=0.1 | Age, sex, BMI, physical activity, smoking, alcohol intake, energy intake, family history of CVD, education level.  All associations remained significant when also adjusting for  #1. Saturated fat, sugar and sodium, healthy dietary pattern  #2. Sugary products, red and processed meat, salty snacks, beverages, and fats and sauces.  #3. Baseline  type 2 diabetes, dyslipidemia, hypertension, and  hypertriglyceridemia and treatments for these conditions. |
| *Zhong et al. 2021* | Prospective Cohort Study  Mean follow-up of 13,5 years | The Prostate, Lung, Colorectal, and Ovarian Cancer Screening Trial, USA  N=91,891 | FFQ  UPF defined by NOVA | UPF (energy adjusted servings/day), divided into quintiles | CVD mortality | Greater UPF intake (5^th^ vs 1^st^ quintile) associated with increased risk of:   - CVD mortality (HR: 1.50; 95% CI: 1.36,1.64, p-trend<0.001) - Heart disease mortality (HR: 1.68; 95% CI: 1.50, 1.87, p-trend<0.001)   Greater effect on CVD mortality and heart disease mortality in women compared to men (P-interaction <0.001).  No association observed for Cerebrovascular disease mortality. | Age, sex, race, educational level, marital status, study  center, aspirin use,  history of hypertension, history of diabetes, smoking, alcohol consumption, BMI, physical activity level, total energy intake |
| *Bonaccio et al. 2021* | Prospective Cohort Study  Median follow-up: 8.2 years | Moli-sani Study  (2005–2010, Italy)  N=22,475 | FFQ  UPF defined by NOVA | UPF (%g/day, divided into quartiles) | - CVD Mortality - Cerebrovascular disease mortality | Greater UPF intake (4^th^ vs 1^st^ quartile) associated with increased risk of:   - CVD mortality (HR: 1.58; 95%CI: 1.23, 2.03), - Cerebrovascular disease mortality   (HR: 1.52; 95% CI: 1.10, 2.09) | Sex, age, energy intake, educational level, housing tenure, smoking, leisure-time physical activity, BMI, history of cancer, CVD, diabetes, hypertension,  hyperlipidemia, residence, Mediterranean Diet Score. |
| *Kim et al. 2019* | Prospective cohort study:  Median follow-up of 19 years | The Third National Health and Nutrition Examination Survey (NHANES III), United States  Representative sample of non-institutionalized adult population  N=11,898 | FFQ  UPF defined by NOVA | UPF (frequency of intake, divided in quartiles) | CVD mortality | UPF intake not associated with CVD mortality (4^th^ vs 1^st^ quartile, HR:1.10, 95%CI: 0.74, 1.67 for; p-trend = 0.86) | Age, sex, race/ ethnicity, total energy intake, poverty level, education level, smoking status, physical activity, and alcohol intake. |
| CVD RISK FACTORS | | | | | | | |
| *Mendonça et al. 2017* | Prospective Cohort Study  Mean follow-up of 9.1 years | The Seguimiento Universidad de Navarra study, Spain  University graduates free of hypertension at baseline  N=14,790 | FFQ  UPF defined by NOVA | UPF (energy adjusted servings/day, divided into tertiles) | Incident hypertension | Higher intake of UPF associated with higher risk of incident hypertension (3^rd^ vs 1^st^ tertile, HR:1.21, 95% CI: 1.06, 1.37; p-trend = 0.004) | Sex, age, physical activity, hours of TV watching, baseline body mass index, smoking status, use of analgesics, following a  special diet at baseline, family history of hypertension, hypercholesterolemia, alcohol consumption, total energy intake, olive oil intake, and  consumption of fruits and vegetables.  Additional adjustment for weight gain during follow-up and sodium intake did not meaningfully alter the association. |
| *Scaranni et al. 2021* | Prospective Cohort Study  4 years of follow-up | The Brazilian Longitudinal Study of Adult Health (ELSA-Brasil), Brazil  Civil servants of Brazilian public academic institutions aged  35-74 years at baseline  N=8,754 | FFQ  UPF defined by NOVA | UPF (%kcal, divided into tertiles) | - Incident hypertension - △ Systolic blood pressure - △ Diastolic blood pressure | Greater UPF intake associated with increased risk of incident hypertension (3^rd^ vs. 1^st^ tertile, OR = 1.23, 95% CI: 1.06-1.44)  UPF intake not associated with incident hypertension when controlling for BMI  UPF intake not associated with changes in diastolic and systolic blood pressure over time. | Age, sex, color or race, education, time since baseline, physical activity, smoking, alcohol consumption, sodium intake, total daily energy intake.  Additionally adjusted for BMI |
| *Monge et al 2021* | Prospective cohort study  Median follow-up of 2.2 years | Mexican Teachers’ Cohort  Disease-free women  aged ≥25 years at baseline  N=64,934 | FFQ  UPF defined by NOVA | Proportion (%) of  energy from total UPF, categorized into: (≤20, 21–25, 26–35, 36–45, >45 %) | Incident hypertension (self-reported) | UPF intake not associated with risk of incident hypertension (IRR: 0.98, 95%CI: 0.84, 1.14 for >45 vs. ≤20%kcal from UPF, p-trend: 0.57). | Indigenous, internet access, insurance, family history of hypertension, menopausal status,  Smoking, physical activity, total energy intake, multivitamin intake. |
| *Duan et al. 2022* | Prospective cohort study  Median follow-up of 41 months | Lifelines cohort study, the Netherlands  35–70 years  N=70,421 | FFQ  UPF defined by NOVA | UPF (% of weight), continuous and divided into quartiles | Incident type 2 diabetes | Greater UPF intake associated with higher risk of developing type 2 diabetes   - per 10% increment in UPF; OR: 1.25 95%CI 1.16, 1.34 - 4^th^ vs 1^st^ quartile: OR: 1.80, 95%CI: 1.47, 2.20   Associations slightly attenuated but still significant when adjusting for BMI | Age, sex, Lifelines diet score, total energy intake, and  alcohol intake, smoking status, educational level, non-occupational  moderate-to-vigorous physical activity level, and TV watching time.  Additionally adjusted for BMI. |
| *Levy et al. 2021* | Prospective cohort study  Median follow-up 5.4 years | UK Biobank  Aged 40-69 at recruitment  N=21,730 | 24-h dietary recall  UPF defined by NOVA | UPF (% of weight), continuous and divided into quartiles | Incident type 2 diabetes | Greater UPF intake associated with greater risk of incident type 2 diabetes:   - Per 10% increment in UPF: HR: 1.12, 95%CI: 1.04, 1.20. - 4^th^ vs 1^st^ quartile: HR:1.44, 95%CI: 1.04, 2.02, p-trend < 0.028. | Age, family history of T2D, stratification by sex and ethnicity, Index of Multiple Deprivation, physical activity, current smoking status, total energy intake, baseline BMI. |
| *Srour et al. 2019 B* | Prospective cohort study  Mean follow-up 6.0 years | The NutriNet-Santé cohort, France  Adult volunteers from general population  N=104,707 | Repeated 24-h recalls  UPF defined by NOVA | UPF (% of weight) | Incident type 2 diabetes | Greater intake of UPF associated with a greater risk of incident type 2 diabetes (HR: 1.15, 95%CI: 1.06, 1.25 per absolute increment of 10% UPF) | Age, sex, education, BMI physical activity, smoking, alcohol, number of 24h recalls, energy intake without alcohol, family history of diabetes, diet quality. |
| *Llavero-Valero et al. 2021* | Prospective cohort study  Median follow-up 12 years | Seguimiento Universidad  de Navarra (SUN) Study  University-graduates  N=20,060 | FFQ  UPF defined by NOVA | UPF (energy-adjusted grams/day), divided into tertiles | Incident type 2 diabetes | Greater intake of UPF (3^rd^ vs 1^st^ tertile) associated with a greater risk of incident type 2 diabetes (HR: 1.53, 95%CI: 1.06, 2.22, p-trend: 0.024).  Additional adjustment for adherence to the Mediterranean diet did not alter the results. | Age, sex, BMI, educational level,  family history of diabetes, smoking status, snacking, 8-item active + sedentary lifestyle score, following a special diet at  baseline.  Additional adjustment for adherence to the Mediterranean diet. |
| *Donat-Vargas et al. 2021* | Prospective cohort study  5-7 years of follow-up | Seniors-Study on Nutrition and Cardiovascular  Risk in Spain (ENRICA) cohort  >60 years at baseline  N=1,082 | Dietary history interview  UPF defined by NOVA | UPF (% of energy) | - Hypertri-glyceridemia - Low HDL - High LDL | Greater intake of UPF (3^rd^ vs 1^st^ tertile) associated with greater odds of:   - Incident hypertri-glyceridemia (OR: 2.66, 95%CI: 1.20, 5.90, P-trend = 0.011) - Incident low HDL (OR: 2.23, 95% CI: 1.22, 4.05; P-trend = 0.012).   UPF consumption not associated with high LDL levels. | Age, sex, total energy intake, education,  marital status, smoking status, BMI, physical  activity, alcohol consumption, fiber intake, number of medications,  number of chronic conditions, intake of minimally processed foods. |

BMI, Body Mass Index; HDL, High-density lipoprotein; HR, Hazard ratio; LDL, Low-density lipoprotein; OR, Odds ratio; RR, Relative risk; UPF, Ultra-processed foods; WC, Waist circumference, 95%CI, 95% confidence interval
