# Supplementary material for: Ultra-processed foods – a scoping review for Nordic Nutrition Recommendations 2023
Source: Food Nutr Res. 2024 Apr 24;68:10.29219/fnr.v68.10616. doi: 10.29219/fnr.v68.10616 (PMC11077402; doi:10.29219/fnr.v68.10616)
Supplement: Supplementary file 5 [file FNR-68-10616-s5.docx]

| Reference | Study design | Population | Dietary assessment | Exposure | Outcomes | Main results | Confounders  adjusted for |
| --- | --- | --- | --- | --- | --- | --- | --- |
| DEPRESSION | | | | | | | |
| *Adjibade et al. 2019* | Prospective cohort study  Mean follow-up 5.4 years | The NutriNet-Santé cohort, France  Adult volunteers from general population (mean age 47.3 at baseline)  N=26,730 | Repeated 24-h recalls  UPF defined by NOVA | UPF (% of weight), continuous and divided into quartiles | Depressive symptoms at ≥2 years after baseline | Greater UPF intake associated with greater risk of incident depressive symptoms:   - A 10% increase in UPF associated with a 21% higher risk of depressive symptoms (HR: 1.21, 95%CI: 0.15, 0.27) - 4^th^ vs 1^st^ quartile: HR:1.30, 95%CI: 1.15, 1.47   Additional adjustment for ‘Healthy’ and ‘Western’ dietary pattern and intakes of lipids, sodium,  and carbohydrates did not alter the associations. | Age, sex, BMI, marital status, educational level, occupational categories, monthly household income, residential area, energy intake without alcohol, number of 24 h records, inclusion month, smoking status, alcohol consumption, and physical activity.  Additional adjustment for ‘Healthy’ and ‘Western’ dietary pattern and intakes of lipids, sodium,  and carbohydrates. |
| *Gómez-Donoso et al. 2020* | Prospective cohort study  Median follow-up 10.3 years | The Seguimiento de Navarra Study, Spain  University graduates (mean age 36.7 at baseline)  N=14,907 | Repeated FFQ  UPF defined by NOVA | UPF (energy adjusted g/day), divided into quartiles | Diagnosis of depression (self-reported) ≥2 years after baseline | Greater UPF intake associated with greater risk of incident depression diagnosis (HR:1.33, 95%CI:1.07, 1.64 for 4^th^ vs 1^st^ quartile; p trend = 0.004) | Sex, BMI, physical activity, smoking status, marital status, living alone,  employment status, working hours per week, health-related career, education, total energy intake, adherence to the Mediterranean diet, and baseline self-perception of competitiveness,  anxiety, and dependence levels. |
| CANCER |  |  |  |  |  |  |  |
| *Fiolet et al. 2018* | Prospective cohort study  Mean follow-up 5.0 years | The NutriNet-Santé cohort, France  Adult volunteers from general population (mean age 42.8 at baseline, 78% females)  N=104,980 | Repeated 24-h recalls  UPF defined by NOVA | UPF (% of weight) | - All cancers - Prostate cancer - Colorectal cancer - Breast cancer | Greater UPF intake associated with higher risk of:   - Overall cancer (HR:1.12, 95%CI: 1.06, 1.18 for a 10% increment in UPF) - Breast cancer (HR: 1.11, 95% CI: 1.02, 1.22 for a 10% increment in UPF).   No statistically significant association observed for prostate cancer or colorectal cancer.  Additional adjustment for intake of fat, sodium, and carbohydrates and Western dietary pattern did not alter the results. | Age, sex, BMI, height, physical activity, smoking, number of 24-hour dietary records, alcohol intake, energy intake, family history of cancer, educational.  Breast cancer analyses were additionally adjusted for number of biological children, menopausal status at baseline,  hormonal treatment for menopause at  baseline and oral contraception use at baseline.  Additional adjustment for intake of fat, sodium, and carbohydrates and Western dietary pattern. |
| FRAILTY |  |  |  |  |  |  |  |
| *Sandoval-Insausti et al. 2020* | Prospective cohort study  Mean follow-up 3.5 years | Seniors-ENRICA Cohort Study, Spain  Adults >59 years  N=1,822 | Diet history  UPF defined by NOVA | UPF (% of energy, divided in quartiles) | Frailty | Greater UPF intake associated with higher risk of incident frailty (4^th^ vs 1^st^ quartile, OR: 3.67, 95%CI: 2.00, 6.73; p-trend <0.001) | Sex, age, education, marital status, smoking, former-drinker status, chronic respiratory  disease, coronary disease, stroke, osteoarthritis/ arthritis, cancer, depression, requiring treatment and number  of medications used. |
| INFLAMMATORY BOWEL DISEASE | | | | | | | |
| *Vasseur et al. 2021* | Prospective cohort study  Mean follow-up 2.3 years | The NutriNet-Santé cohort, France  Adult volunteers from general population (mean age 42.8 at baseline)  N=105,832 | Repeated 24-h recalls  UPF defined by NOVA | UPF (% of weight, divided in tertiles) | Inflammatory bowel disease | UPF intake not associated with inflammatory bowel disease | Age, sex, income, education, marital status, residence, BMI, physical activity, smoking, hormonal contraception, number of 24-hour dietary records, energy intake. |
| *Narula et al. 2021* | Prospective cohort study  Median follow-up 9.7 years | The Prospective Urban Rural Epidemiology (PURE) cohort  Adults aged 35-70 years in 21 low-, middle-, and high-income countries  N=116,087 | FFQ  UPF defined by NOVA | UPF intake, categorized as: <1 serving/day, 1-4 servings/day, or ≥5  servings/day | Inflammatory bowel disease | Higher intake of UPF associated with higher risk of incident IBD (HR: 1.82, 95%CI: 1.22, 2.72 for ≥5 servings/day and HR: 1.67, 95%CI: 1.18, 2.37 for 1-4 servings/day, vs. <1 serving/day, resp., p-trend=0.006). | Age, sex, geographical region, education, alcohol intake, smoking, BMI, total energy intake, location. |
| OTHER DISEASES | | | | | | | |
| *Zhang et al. 2021* | Prospective cohort study  Mean follow-up 4.2 years | Tianjin Chronic Low-grade Systemic Inflammation and  Health cohort study, China  Adults aged 18-90 years  N=18,444 | FFQ  UPF defined by NOVA | UPF (g/day) | Hyperuricemia | Greater UPF intake associated with higher risk of  hyperuricemia (4^th^ vs. 1^st^ quartile, HR:1.16, 95%CI: 1.05, 1.28, p-trend = 0.02) | Sex, age, BMI, smoking, alcohol consumption,  education, employment, household income, physical activity, family history of disease (CVD, hypertension, hyperlipidemia,  and diabetes), depressive symptoms, metabolic syndrome, hypertension, hyperlipidemia, diabetes, total energy intake, dietary patterns, baseline serum uric acid and glomerular filtration rate. |
| Rey-García et al. 2021 | Prospective cohort study | Seniors-ENRICA-1 cohort, Spain  Adults>59y  N=1,312 | Dietary history (DH-ENRICA)  UPF defined by NOVA | UPF (%energy and g/day/kg body weight, divided into tertiles) | Renal  function decline | Greater UPF intake associated with higher risk of renal function decline:   - 3rd vs 1st tertile of %energy, OR: 1.74, 95%CI: 1.14, 2.66, p-trend = 0.026 - 3rd vs 1st tertile of g/kg/day, OR:1.62, 95%CI: 1.06, 2.49, p-trend = 0.043 | Sex, age, total energy intake, education, smoking, former-drinker status, physical activity,  time spent watching TV, fiber intake, number of chronic conditions, number of medications  used per day, hypertension, diabetes, hyper-cholesterolemia, BMI. |

BMI, Body mass index; CVD, Cardiovascular disease; HR, hazard ratio; OR, odds ratio; UPF, Ultra-processed food; 95%CI: 95% confidence intervals
