# Supplementary material for: Ultra-processed foods – a scoping review for Nordic Nutrition Recommendations 2023
Source: Food Nutr Res. 2024 Apr 24;68:10.29219/fnr.v68.10616. doi: 10.29219/fnr.v68.10616 (PMC11077402; doi:10.29219/fnr.v68.10616)
Supplement: Supplementary file 6 [file FNR-68-10616-s6.docx]

| Reference | Study design | Population | Dietary assessment | Exposure | Outcomes | Main results | Covariates |
| --- | --- | --- | --- | --- | --- | --- | --- |
| *Rohatgi et al. 2017* | Prospective cohort study | Pregnant women (N=45) and their infants, United States | FFQ | 3^rd^ trimester UPF intake (% of energy) | Mother:  Gestational weight gain, triglycerides, fasting insulin/glucose, c-reactive protein, free fatty acids  Infant:  thigh skinfold, subscapular skinfold, total body adiposity, fasting insulin/glucose, c-reactive protein, free fatty acids | Greater UPF intake associated with greater:   - Gestational weight gain (β=1.3, 95%CI: 0.3, 2.4) - Infant thigh skinfold (β=0.3, 95%CI: 0.005,0.4) - Infant subscapular skinfold (β=0.1, 95%CI: 0.02, 0.3) - Infant total body adiposity (β=0.6, 95%CI: 0.04, 1.2)   UPF intake not associated with maternal triglycerides or maternal/infant fasting insulin/glucose, c-reactive protein, free fatty acids | Maternal outcomes: age, race, weight status, SES, total energy and fat intake, physical activity.  Infant outcomes: gestational age,  maternal age,  race, weight status, SES, total energy and fat intake and physical activity. |
| *Gomes et al. 2021* | Prospective cohort study | Pregnant women from São Paulo, Brazil  N=259 | 24-h recall  UPF defined by NOVA | Pregnancy UPF intake (% of energy) | Weight gain (g) within trimester | A 1% point increase in UPF was associated with a 4.17g (95 % CI 0.55, 7.79) increase in weekly GWG during the 3^rd^ trimester.  No association in the 2^nd^ trimester. | Education, parity, skin color, pre-gestational BMI |
| *Leone et al. 2021* | Prospective cohort study | Seguimiento Universidad  de Navarra (SUN) Study, Spain  University-graduates  N=3,730 women | FFQ | Pre-pregnancy UPF intake (frequency of consumption, divided into tertiles) | Gestational diabetes | No association in overall sample.  In age-stratified analyses,  UPF intake was associated with higher risk of GD in women aged 30+ y (OR:2.05, 95%CI: 1.03, 4.07) but not in younger women. | Age, BMI, education, smoking status, physical  activity, family history of diabetes, recruitment year, time between recruitment and the first pregnancy  or GDM, number of pregnancies during follow-up, parity, multiple pregnancies, time spent  watching TV, hypertension, following a nutritional therapy and energy intake |
| *Borge et al. 2021* | Prospective cohort study  Registry data  8 years of follow-up | The Mother, Father and Child Cohort Study, Norway  Mothers & their children  N=37,787 (symptoms analysis)  N= 77,768 (diagnosis analysis) | FFQ  UPF defined by NOVA | UPF index during pregnancy (% of energy) | ADHD symptoms at 8 years (reported by mothers)  ADHD diagnoses at 8 years (from registry) | Higher UPF index in pregnancy was associated increased ADHD symptoms score at age 8 years (AME: 0.25, 95%CI: 0.13, 0.38)  Maternal UPF index in pregnancy was not associated with the child’s relative risk of ADHD diagnosis at age 8 years (AME:1.07, 95%CI: 0.90, 1.08) | Maternal pre-pregnancy BMI, maternal education, smoking and alcohol intake during  pregnancy, maternal symptoms of depression and ADHD, maternal age, parity, child sex, child diet and childbirth quarter. |

ADHD, Attention Deficit Hyperactivity Disorder; AME, Average marginal effect; BMI, Body Mass Index; OR, Odds ratio; 95%CI, 95% Confidence interval.
