# Supplementary material for: Ultra-processed foods – a scoping review for Nordic Nutrition Recommendations 2023
Source: Food Nutr Res. 2024 Apr 24;68:10.29219/fnr.v68.10616. doi: 10.29219/fnr.v68.10616 (PMC11077402; doi:10.29219/fnr.v68.10616)
Supplement: Supplementary file 7 [file FNR-68-10616-s7.docx]

| Reference | Study design | | Population | Dietary assessment | | Exposure | Outcomes | Main results | Confounders  adjusted for | | |
| --- | --- | --- | --- | --- | --- | --- | --- | --- | --- | --- | --- |
| EXCESS WEIGHT, WEIGHT GAIN & ADIPOSITY | | | | | | | | | | | |
| *Costa et al. 2019* | Prospective secondary analyses based on a RCT (dietary counseling on breastfeeding and dietary practices)  4 years of follow-up | | Mothers and children from a low-income population, Brazil  N=307 | Repeated 24-h recalls (2) at age 4 and 8 years  UPF defined by NOVA | | UPF (% of energy) | - △ BMI (kg/m^2^) - △ WC (cm) - △ Waist to height ratio (cm) - △ Skinfold sum (mm) | A 10% absolute increase in energy consumption from UPF at age four was associated to a 0.7 cm greater increase in WC between age 4 and age 8 (97%CI: 0.1-0.13).  No significant association for BMI, waist to height ratio nor sum of skinfolds. | Sex, group status in the early phase (intervention  and control), maternal pre-pregnancy BMI, birth weight, breastfeeding, family income, maternal education, total screen time. | | |
| *Chang et al. 2021* | Prospective cohort study  Children were followed up from 7 to 24 years of age  Median follow up 10.2 years | | Avon Longitudinal Study of Parents and Children (ALSPAC)  N=9025 | Food diaries (3 days)  UPF defined by NOVA | | UPF (% of weight, divided into quintiles) | Trajectories of adiposity outcomes:  BMI, fat mass index (FMI), total fat %, lean mass index (LMI), weight, WC | UPF intake associated with greater annual increase (5^th^ vs 1^st^ quintile):   - BMI, β: 0.06 (95%CI, 0.04-0.08) - FMI, β: 0.03, (95%CI, 0.01-0.05) - Weight, β: 0.20 95%CI, 0.11-0.28 - WC, β: 0.17 (95% CI: 0.11-0.22)   UPF not associated with total fat % or LMI. | Sex, race, birth weight, physical activity,  Index of Multiple Deprivation, mother’s pre-pregnancy BMI, marital status,  highest educational attainment,  socioeconomic status, and child’s total energy intake at baseline. | | |
| *Costa et al. 2021* | Prospective cohort study | | Pelotas-Brazil 2004 Birth Cohort  N=3,454 | FFQ  UPF defined by NOVA | | UPF (g/day) | Change in fat mass index (FMI) from 6y to 11y of age | Greater UPF intake associated with greater increase in FMI (per 100g/day increment, β:0.14, 95%CI: 0.13, 0.15).  Adjustment for total energy intake attenuated association, but it remained statistically significant (β:0.05, 95%CI: 0.04, 0.06). | Skin color, maternal age and schooling, birthweight, sex, screen time, energy intake/ expenditure ratio, grams from other food sources than UPF  Additional adjustment for total energy intake. | | |
| *Vedovato et al. 2021* | Prospective cohort study  Follow-up: 6 years (from age 4 to 10) | | Generation XXI, Portugal  N=1,175 | Portuguese Children’s Eating Behavior Questionnaire  (P-CEBQ)  UPF defined by NOVA | | UPF (%kcal/d) | BMI z-score | UPF at 4 years significantly  associated with BMI z-score at age 10 (β: 0.028, 95%CI: 0.006, 0.051)  UPF at 7 years not associated with BMI z-score at age 10. | Maternal age, education and BMI before pregnancy and child exclusive breast-feeding for the first 6 months, practice of physical exercise and  daily screen time and BMI z-score at 4y. | | |
| CARDIOVASCULAR RISK FACTORS | | | | | | | | | | |  |
| *Costa et al. 2019* | | Prospective secondary analyses based on an RCT (dietary counseling on breastfeeding and dietary practices)  4 years of follow-up | Children from a low-income population  N=307  Brazil | | Repeated 24-h recalls (2) at age 4 and 8 years  UPF defined by NOVA | UPF (%kcal) at age 4 | Parameters at age 8:   - Glucose (mmol/l) - Insulin (uU/ml) - HOMA-IR | UPF intake at age 4 years not associated with glucose, insulin or HOMA-IR at age 8. | | Sex, group status in the early phase (intervention  and control), maternal pre-pregnancy BMI, birth weight, breastfeeding, family income, maternal education, total screen time. |  |
| *Leffa et al. 2020* | | Prospective secondary analyses based on an RCT (dietary counseling on breastfeeding and dietary practices)  3 years of follow-up | Children from a low-income population, Brazil  N=308 | | Repeated 24-h recalls  UPF defined by NOVA | UPF (%kcal divided into tertiles) | - Total cholesterol - HDL-cholesterol - Triglycerides | Higher UPF intake at age 3 years was associated with higher levels of total cholesterol and triglycerides at age 6 years.  Per absolute increment of 10% UPF:   - Total cholesterol, β: 0.07 mmol/l, 95%CI: 0.00, 0.14 - Triglycerides, β: 0.04 mmol/l, 95%CI: 0.01, 0.07   3^rd^ vs 1^st^ tertile:   - Total cholesterol β: 0.22 mmol/l; 95%CI: 0.04, 0.9 - Triglycerides β:0.11 mmol/l, 95%CI: 0.01, 0.20)   UPF intake at age 3 years not associated with HDL-cholesterol at age 6 years. | | Sex, group status in the early phase (intervention and control), family income, maternal pre-pregnancy BMI, child birth weight, BMI z-scores at 3 years, intakes of total energy and total fat at age 3 years. |  |
| *Rauber et al. 2015* | | Prospective secondary analyses based on an RCT (dietary counseling on breastfeeding and dietary practices)  4 years of follow-up | Children from a low-income population, Brazil  N=345 | | Repeated 24h-recalls  UPF defined by NOVA | UPF (%kcal) | - △ Total cholesterol - △ LDL-cholesterol - △ HDL-cholesterol - △ nHDL-cholesterol - △ Tri-glycerides | Greater UPF intake at age 3-4 years associated with higher increase in and total cholesterol (β:0.430; p = 0.046) and LDL cholesterol (β:0.369; p = 0.047) from age 3-4 to 7-8 years.  UPF intake at 3-4 years was not associated with HDL-cholesterol, nHDL cholesterol or triglycerides at age 7-8 years. | | Sex, group status in the early phase (intervention and control), birth weight, family income, maternal education, and BMI for-  age z-scores and total energy intake at 7-8 years |  |
| ASTHMA | | | | | | | | |  | | |
| *Machado Azeredo et al. 2020* | Prospective cohort study  5 years of follow-up | | The 2004 Pelotas Birth Cohort Study, Brazil  Children (6 years old at baseline)  N=2,190 | FFQ  UPF defined by NOVA | | UPF intake (% of energy, divided in quintiles) | - Wheeze - Asthma - Severe asthma | There was no association between UPF intake at 6 years and wheeze, asthma or severe asthma at age 11 | Intake adjustments, total energy intake, family income, maternal education, maternal age, maternal skin color, parity,  smoking during pregnancy, maternal asthma, child´s sex, parental smoking. | | |

BMI, Body Mass Index; HDL, High-density lipoprotein; nHDL, Non-high-density lipoprotein; LDL, Low-density lipoprotein; UPF, Ultra-processed foods; WC, Waist circumference, 95%CI, 95% confidence interval
